# Therapeutic role of granulocyte colony-stimulating factor (G-CSF) for infertile women under in vitro fertilization and embryo transfer (IVF-ET) treatment: a meta-analysis

**DOI:** 10.1007/s00404-018-4892-4

**Published:** 2018-09-15

**Authors:** Ling Zhang, Wei-Hai Xu, Xiao-Hua Fu, Qiong-Xiao Huang, Xiao-Yan Guo, Lin Zhang, Shi-Shi Li, Jing Zhu, Jing Shu

**Affiliations:** Department of Reproductive Endocrinology, Hangzhou Medical College, Zhejiang Provincial People’s Hospital, 158 Shangtang Road, Hangzhou, China

**Keywords:** Granulocyte colony-stimulating factor, Embryo transfer, Infertility, Meta-analysis, Repeated implantation failure, Thin endometrium

## Abstract

**Objective:**

The aim of this meta-analysis is to explore the beneficial role of granulocyte colony-stimulating factor (G-CSF) on infertile women under artificial reproduction technology treatment.

**Method:**

Medline, Embase and ISI Web of Science databases were searched to identify relevant randomized control trials. Studies before July, 2017 were included for primary screening. Meta-analysis of the total and subgroup patients was conducted, and relative risks (RRs) and their 95% confidence intervals (95% CI) were calculated by a fixed-effect model if no heterogeneity (evaluated as* I*^2^ statistic) existed. Otherwise, a random-effects model was adopted. Subgroup analysis was performed by administrating route or clinical indication. Egger test and influence analysis were conducted to evaluate the publication bias and study power, respectively.

**Results:**

The final selection enrolled 10 RCTs, involving 1016 IVF-ET cycles (521 distributed to the G-CSF group and 495 to the control). Compared with control group, G-CSF administration could significantly improve clinical pregnancy rate (CPR, RR 1.89, 95% CI 1.53–2.33), while it had no beneficial effect on embryo implantation rate (IR, RR 1.84, 95% CI 0.84–4.03). The subgroup analysis by administration route showed that both uterine infusion and subcutaneous injection can produce a substantial increase in CPR, with the pooled RRs (95% CI) 1.46 (1.04–2.05) and 2.23 (1.68–2.95), respectively. Nevertheless, most of included RCTs dealt with the RIF subjects, and the pooled analysis of this data showed a higher PR and IR in G-CSF group as compared to that in the control, with the RRs (95% CI) 2.07 (1.64–2.61) and 1.52 (1.08–2.14), respectively. Egger regression test did not demonstrate any significance for the publication bias.

**Conclusion:**

G-CSF administration has a beneficial role on the clinical outcome after embryo transfer by both routes of local infusion and systematic administration, especially for the cases with RIF. Further RCTs are needed to investigate the role of G-CSF in thin endometrium patients.

**Electronic supplementary material:**

The online version of this article (10.1007/s00404-018-4892-4) contains supplementary material, which is available to authorized users.

## Introduction

Implantation of a competent blastocyst into receptive endometrium is key to build a successful pregnancy [1]. Despite major advancement in reproductive medicine over the last few decades, implantation failure still makes frequent appearance during the process of assisted reproductive technology (ART) [2, 3]. Repeated implantation failure (RIF), generally defined as failure of three in vitro fertilization and embryo transfer (IVF-ET) cycles in which one or two high-grade quality embryos were transferred to the patient in each cycle [4], represents an enormous emotional and financial burden for the patient. Poor endometrial receptivity has been generally considered as a major cause of the failure of embryo implantation, and endometrial thickness as an important component of endometrial receptivity [5]. Several therapies have been proposed for solving the problem in endometrial receptivity, such as extended estrogen administration, treatment with low-dose aspirin, vaginal sildenafil citrate, and treatment with pentoxifylline and tocopherol, and proven successful in some cases. However, many cases still remain resistant to these treatments [5].

Successful embryo implantation requires an intricate biological interaction between the implanting embryo and the host endometrium [6]. A bulk of molecular factors have been implicated in this complex process, including endometrial integrins, extracellular matrix molecules, adhesion molecules, growth factors, and ion channels [1]. Granulocyte colony-stimulating factor (G-CSF) belongs to the family of colony-stimulating factors (CSF) synthesized by multiple cell types (e.g., endothelial cells, fibroblasts, macrophages, lymphocytes) [7, 8], and has been proven to originate from some reproductive tissue cells as well, such as human ovary [9] and endometrium [10]. Particularly, some pieces of evidence have showed that G-CSF or its receptor be located in luteinized granulosa cells, placenta trophoblastic cell and oocytes [11–13]. Currently, several physiological roles have been suggested for G-CSF during the process of pregnancy forming, i.e., promoting embryo cleavage and blastocyst formation [13], regulating endometrial expressions crucial for a series of implantation processes including endometrial vascular remodeling, local immune modulation and cellular adhesion pathways [14], and targeting follicle development and ovulation [15].

The therapeutic effect of G-CSF in patients with RIF has been investigated as early as 2000 by Würfel and the colleagues, and the results show that systematic administration of G-CSF is able to enhance the implantation rate dramatically [16]. Since then, bulks of similar studies have been conducted for RIF cases due to poor endometrial thickness or other reasons, but the conclusions are inconsistent. Even in rigorously randomized control trials (RCTs), only about half reach a conclusion that G-CSF can improve the endometrium thickness, implantation rate or clinical pregnancy rate after IVF treatment [16–18], while the remains negative [19–23]. This inconsistence might be owing to the heterogeneity in administration route or clinical conditions between studies. Indeed, currently published studies were structured into various designs, such as randomized control trials (RCTs), observational studies, self-controlled trials or single arm studies; included subjects of different clinical conditions including RIF, thin endometrium or unselected patients, and did not adapt the same administration route, systematic injection or intrauterine infusion.

Single study may be limited by sample size, research design, administration route, clinical conditions, or patient’s ethnicity and age, and underpowered to achieve a comprehensive and reliable conclusion. Meta-analysis has the benefit to overcome this limitation by increasing the sample size. Therefore, this study was designed to explore the efficacy of G-CSF on infertile patients undergoing IVF-ET treatment with RIF.

## Methods and procedures

The review protocol was registered in PROSPERO (CRD42018056662). Randomized controlled trials (RCTs) comparing G-CSF treatment versus the control were included in this meta-analysis. Pseudo-randomized trials were excluded.

We collected the relevant studies by searching the databases of Cochrane Central Register of Controlled Trials (CENTRAL), Medline, Embase and ISI Web of Science updated in July, 2017, using the keywords: (‘Granulocyte colony stimulating factor’ OR ‘Granulocyte Colony-Stimulating Factor’ OR ‘G-CSF’ OR ‘CSF’) AND (‘Assisted Reproductive Techniques’ OR ‘ART’ OR ‘In Vitro Fertilization’ OR ‘IVF’ OR ‘Intracytoplasmatic Sperm Injection’ OR ‘ICSI’OR ‘embryo transfer’ OR ‘FET’). There were no limitations on the type of the publication. All languages were accepted. We also searched for study protocols and ongoing trials in ClinicalTrials.gov (https://clinicaltrials.gov/). References of retrieved articles were also screened.

**Fig. 1:**
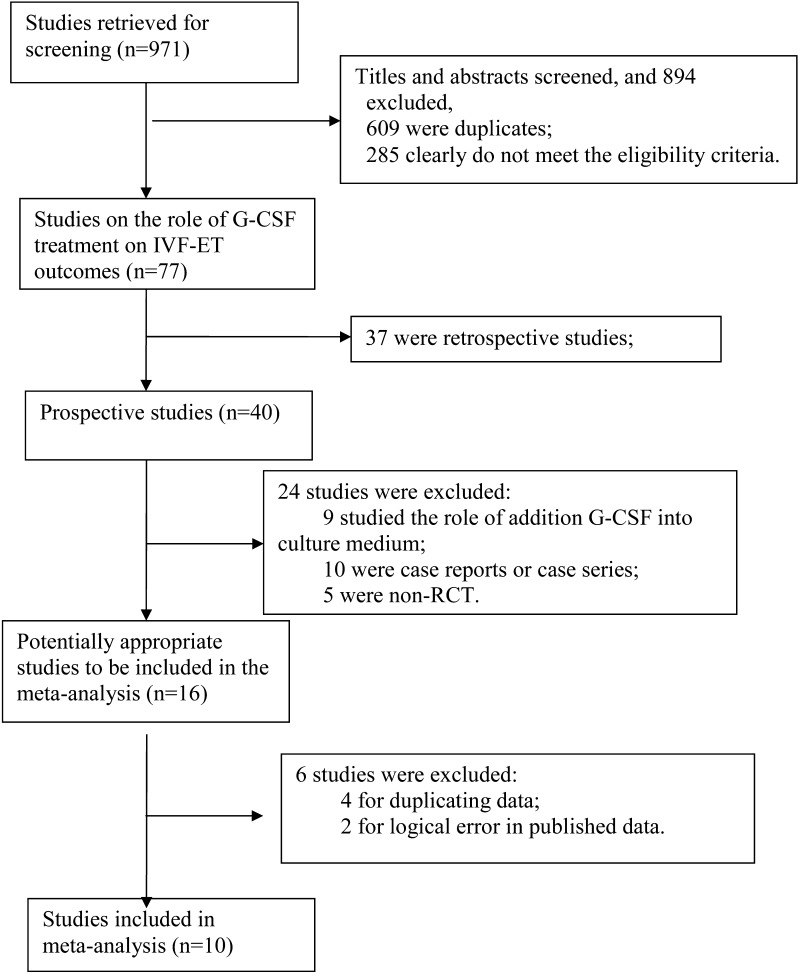
Flowchart of the study selection process

Our primary outcome measure was clinical pregnancy rate (CPR) per woman randomly assigned, and the secondary one implantation rate (IR) per embryo transferred. All literatures were reviewed independently by two authors. The flow chart for study selection was shown in Fig. 1. Two authors extracted data independently and in duplicate, and reached on all items including author’s last name, journal and year of publication, country of origin, ethnicity of the patients, definition of RIF or thin endometrium, count of each event in GSF group and the control. The results were compared and disagreements were discussed and resolved with consensus.

Risk of bias in individual study was structured using the Cochrane risk of bias assessment tool to assess: selection (random sequence generation and allocation concealment); performance (blinding of participants and personnel); detection (blinding of outcome assessors); attrition bias (incomplete outcome data); reporting bias (selective outcome reporting) and other potential sources of bias. The trials were classified as being at ‘low’, ‘high’ or ‘unclear’ risk of bias.

The pooled RRs and their 95% confidence interval (CI) were estimated to assess the role of G-CSF treatment on the outcomes of IVF-ET. The pooled RRs were calculated through a Mantel–Haenszel fixed effects model if there was no heterogeneity. Otherwise, a random-effects model was adopted. Subgroup analysis was performed by administration route and clinical indications. Statistical heterogeneity across studies was formally tested using Cochran’s test. The *I*^2^ statistic was examined and *I*^2^ > 50% was considered significant for the heterogeneity between studies. An influence analysis was conducted to describe how robust the pooled estimator is after removal of individual studies. An individual study was suspected of excessive influence if the point estimate of its omitted analysis lies outside the 95% CI of the combined analysis. Publication bias across studies was assessed using the Egger regression test and Begg’s funnel plot. All analyses were conducted in Stata software (Version 14.0; Stata Corporation, College Station, TX, USA).

## Results

### Study characteristics

The last electronic search was conducted in July 25, 2017, retrieving a total of 971 records. After screening the titles and abstracts, we removed 894 records including 609 duplicates and 285 ones that did not meet the eligibility criteria. A total of 77 records were further examined for eligibility, and 61 removed for non-RCT design (*n* = 52) or non-maternal administration route (*n* = 9). Finally, 16 articles were included into the stage of data extraction, then 4 excluded for duplicating data [24–27], 2 for logical error in published data [28, 29] and 1 without data regarding the IVF outcome. Finally, a total of ten studies were included in this meta-analysis [16–23, 30, 31].

With these ten articles published between 2000 and 2016, 1036 IVF-ET patients in all were randomized, from a wide range of regions including Europe [16, 18, 21, 31], North America [19] and Asia [17, 20, 22, 23, 30]. Among all studies, one evaluated the role of G-CSF treatment for unselected patients [19], one for the cases with thin endometrium [20], and remaining eight for those with RIF [16–18, 21–23, 30, 31]. The detailed characteristics of these studies are shown in Table 1.

**Table 1:** Characteristics of the studies included in the meta-analysis

Author, year	Country	Period of enrolment	Publication type	Sample size	Female age (years) CSF/control	Indications	Diagnostic criteria	Inclusion and exclusion criteria
Aleyasin A, 2016 [25]	Iran	March 2015–January 2016	Origin article	112	33.5 ± 4.2/32.4 ± 5.2	RIF	Transferred embryos fail to implant after three IVF cycles	< 40 years, with normal endometrium thickness, and without sensitivity of GCSF, or any systemic disease, or detection of Asherman’s syndrome, fibroids and/or polyps
Barad DH, 2014 [19]	USA	October 2010–January 2013	Origin article	141	39.79 ± 5.13/39.38 ± 6.03	Unselected	–	With normal endometrial thickness, and without renal disease, sickle cell disease, or a history of malignancy
Davari-Tanha F, 2016 [23]	Iran	December 2011–January 2014	Origin article	80	35.5 ± 4.32/35.3 ± 3.98	RIF	History of three times implantation failure with at least four good-quality embryos	< 40 years, without uterine, thrombophilic factors, or history of renal disease, sickle cell disease or malignancy or sensitivity of G-CSF
Eftekhar M, 2016 [22]	Iran	October 2014–February 2015	Origin article	90	32.55 ± 4.61/31.75 ± 5.16	RIF	At least two implantation failures	20–40 years, without sickle cell disease, chronic neutropenia, malignancy history, renal failure, congenital fructose intolerance, respiratory infection, endometriosis, or sever male factor
Kim CH, 2011 [30]	South Korea	NR	Meeting abstract	82	NR	RIF	At least three previous failed IVF attempts with good quality embryos	29–40 years, without thrombophilia or anatomic abnormalities of uterine cavity
Obidniak D, 2016, part I [21]	Russia	NR	Meeting abstract	130	NR	RIF	At least two previous failed IVF attempts with good-quality blastocysts	32–40 years, with normal endometrial thickness and availability of vitrified blastocyst, without congenital uterine anomalies or Asherman’s syndrome
Obidniak D, 2016, part II [21]	Russia	NR	Meeting abstract	130	NR	RIF	At least two previous failed IVF attempts with good-quality blastocysts	32–40 years, with normal endometrial thickness and availability of vitrified blastocyst, without congenital uterine anomalies or Asherman’s syndrome
Scarpellini F, 2011 [31]	Italy	NR	Meeting abstract	89	NR	RIF	NR	NR
Scarpellini F, 2012 [18]	Italy	January 2008–December 2010	Meeting abstract	109	NR	RIF	At least three previous failed IVF attempts with at least seven good embryos	< 39 years, absence of systemic diseases
Singh R, 2015 [20]	India	January 2014–December 2014	Meeting abstract	48	< 40	Thin endometrium	NR	< 42 years
Würfel W, 2000 [16]	Germany	NR	Meeting abstract	138	NR	RIF	At least three previous failed IVF attempts with at least five embryos and more than 50% embryos transferred in good quality	< 40 years, undergoing the fourth or fifth IVF or ICSI cycle

Author, year	Drug route/dose	Administration time	Description of the control	Protocol	Embryo age	Previous failure CSF/control	Embryos transferred CSF/control	Good embryos transferred (%) CSF/control
Aleyasin A, 2016 [25]	Subcutaneously 300 μg	1 h before the ET	No therapy	Fresh ET	D3	3/3	2.3 ± 0.6/2.5 ± 0.6	NR
Barad DH, 2014 [19]	Intrauterine infusion 300 µg	The morning of hCG administration	Normal saline	Fresh or frozen ET	D3 or D5	NR	2.41/2.47	45.57/39.29
Davari-Tanha F, 2016 [23]	Intrauterine infusion 300 μg	At the time of oocyte retrieval. In FET cycle, at the day of starting progesterone	Normal saline	Fresh or frozen ET	D3 or D5	3.5 ± 2.1/4.2 ± 1.5	NR	NR
Eftekhar M, 2016 [22]	Intrauterine infusion 300 μg	At the time of oocyte retrieval	Without GSF infusion	Fresh ET	D3	2.57 ± 1.69/3.41 ± 1.54	2.11/2.35	61.4/57.80
Kim CH, 2011 [30]	NR 100 µg × 2	The day of ET and the fourth day after ET	–	Fresh ET	D3	NR	NR	NR
Obidniak D, 2016, part I [21]	Intrauterine infusion 300 μg	5 days prior to ET, or at the day of ET	No therapy	Frozen ET	Blastocyst	NR	NR	NR
Obidniak D, 2016, part II [21]	Subcutaneously 300 μg	5 days prior to ET, or at the day of ET	No therapy	Frozen ET	Blastocyst	NR	NR	NR
Scarpellini F, 2011 [31]	Subcutaneously 1.5 mg/kg/day	From the day of transfer to the day of hCG test, and if it was positive the treatment was continued for other 40 days	Normal saline	Fresh ET	NR	NR	NR	NR
Scarpellini F, 2012 [18]	Subcutaneously 60 mg/daily	From the day of transfer to the day of hCG test, and if it was positive the treatment was continued for other 40 days	Normal saline	NR	NR	NR	NR	NR
Singh R, 2015 [20]	Intrauterine infusion 300 µg or 300 µg × 2	The morning of hCG administration	Normal saline	Fresh ET	NR	NR	NR	NR
Würfel W, 2000 [16]	Systematically 300 μg	On the day of ET	–	NR	NR	NR	NR	NR

### Meta-analysis

**Fig. 2:**
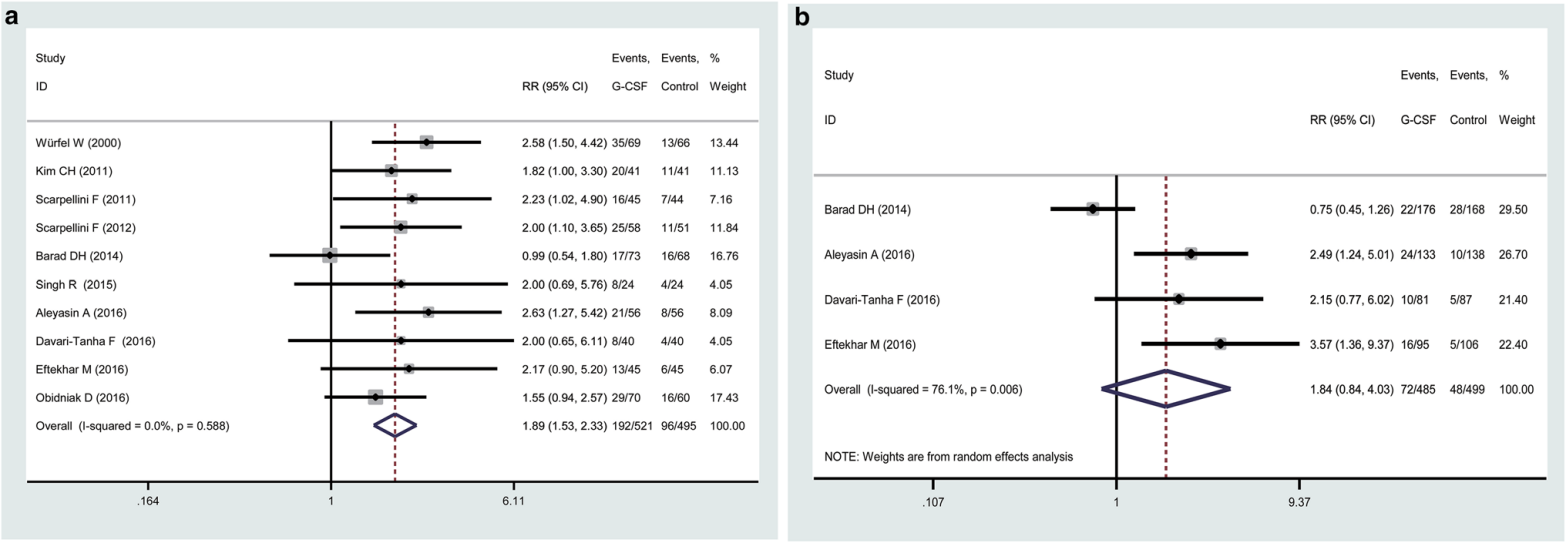
Forest plot comparing the effect of G-CSF on CPR and IR in infertile women undergoing IVF/ICSI. The forest plot shows a benefit of G-CSF administration for CPR (**a**), but none for IR (**b**). A random-effects model was used for IR analysis because the included studies had substantial between-study heterogeneity. Horizontal lines indicate 95% CIs; boxes show the study-specific weight; diamond represents combined effect size; dashed line indicates the overall estimate

Ten studies all described the role of studied administration on CPR after ART. Figure 2a showed the forest plots RRs on CPR, and the pooled RR value was 1.89 (95% CI 1.53–2.33, *P* = 0.00), indicating that G-CSF treatment may be beneficial to improve CPR in IVF-ET patients. Between studies homogeneity has been identified, as judged by the value *I*^2^ (0.0%). Four studies reported the data of embryo implantation [19, 22, 23, 25], and the pooled analysis did not show any beneficial effect of G-CSF treatment (RR 1.84, 95% CI 0.84–4.03, *P* = 0.13) (Fig. 2b). The between-study variance was relatively high in this analysis (*I*^2^ = 76.1%). However, limited reports included in our study make it impossible to further examine the variance factors, such as mete-regression analysis.

Egger regression test of the data of CPR and IR did not find any significance (*P* = 0.45 and 0.24, respectively) and Begg’s funnel plot showed an evident balance, indicating a low chance of publication bias (Fig. S1). Figure S2 presents the result of influence analysis after removal of individual studies, and none individual study was found to excessively influence the pooled effect for both CPR and IR analyses (Table 2).

**Table 2:** Judgements about risk of bias of included study

Study	Selection bias		Performance and detection bias		Attrition bias		Selective reporting		Other bias	
	Risk	Explanation	Risk	Explanation	Risk	Explanation	Risk	Explanation	Risk	Explanation
Aleyasin A, 2016 [25]	L	A computer-generated randomization table was used.	H	No blinding	L	No loss of follow-up	L	Not suspected	L	None
Barad DH, 2014 [19]	L	A computer-generated randomization table was used	L	Study group was blinded to patients, physicians and nurses	L	No loss of follow-up	L	Not suspected	L	None
Davari-Tanha F, 2016 [23]	L	A computer-generated randomization table was used	L	Study group was blinded to patients and clinician	L	No loss of follow-up	L	Not suspected	L	None
Eftekhar M, 2016 [22]	L	Randomization with enveloped pocket method	H	No blinding	L	No loss of follow-up	L	Not suspected	L	None
Kim CH, 2011 [30]	U	Method of random sequence allocation was not described	U	Method of blinding was not described	L	No loss of follow-up	H	Did not report implantation rate	H	Meeting abstract
Obidniak D, 2016, part I [21]	U	Method of random sequence allocation was not described	U	Method of blinding was not described	L	No loss of follow-up	H	Did not report implantation rate	H	Meeting abstract
Singh R, 2015 [20]	L	A computer-generated randomization table was used	U	Method of blinding was not described	L	No loss of follow-up	H	Did not report implantation rate	H	Meeting abstract
Scarpellini F, 2011 [31]	U	Method of random sequence allocation was not described	U	Method of blinding was not described	L	No loss of follow-up	H	Did not report implantation rate	H	Meeting abstract
Scarpellini F, 2012 [18]	U	Method of random sequence allocation was not described	U	Method of blinding was not described	L	No loss of follow-up	H	Did not report implantation rate	H	Meeting abstract
Würfel W, 2000 [16]	U	Method of random sequence allocation was not described	U	Method of blinding was not described	L	No loss of follow-up	H	Did not report implantation rate	H	Meeting abstract

*U* unclear, *H* high, *L* low

**Fig. 3:**
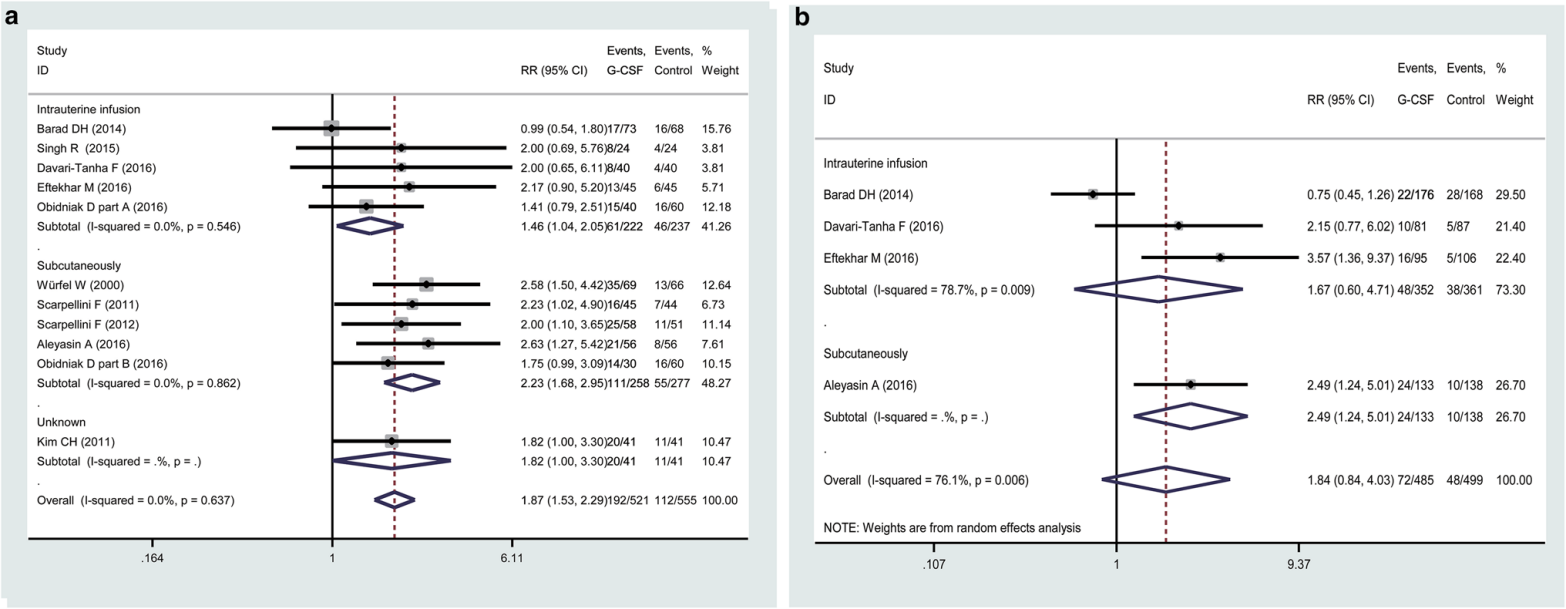
Forest plot comparing the effect of G-CSF on CPR (**a**) and IR (**b**) in infertile women undergoing IVF/ICSI for different routes of administration. Horizontal lines indicate 95% CIs; boxes show the study-specific weight; diamond represents combined effect size; dashed line indicates the overall estimate

Subgroup analysis was further carried out according to the route of G-CSF administration (subcutaneous injection *n* = 5, uterine infusion *n* = 5, and unknown = 1) and the indications of G-CSF administration (unselected fertility *n* = 1, thin endometrium *n* = 1, and RIF *n* = 8). In the subgroup analysis by administration route, we found an increased CPR for both uterine infusion and subcutaneous injection, and the pooled RRs (95% CI) were 1.46 (1.04–2.05) and 2.23 (1.68–2.95), respectively (Fig. 3a). Among the studies reporting the outcome of IR, four focused on the routes of uterine infusion, and only one on the subcutaneous injection. The subgroup analysis has failed to find an increased IR after G-CSF treatment via uterine infusion (Fig. 3b). The only study with route of subcutaneous injection identified a beneficial role of G-CSF on IR [17].

In the subgroup analysis by the indications of G-CSF administration, a higher PR and IR has been found for G-CSF group as compared to the control after pooled analysis of RIF subjects, with the RRs (95% CI) 2.07 (1.64–2.61) and 1.52 (1.08–2.14), respectively. For unselected fertility or thin endometrium, only one study has been reported, and none improved PR or IR suggested for any population [19, 20].

## Discussion

**Fig. 4:**
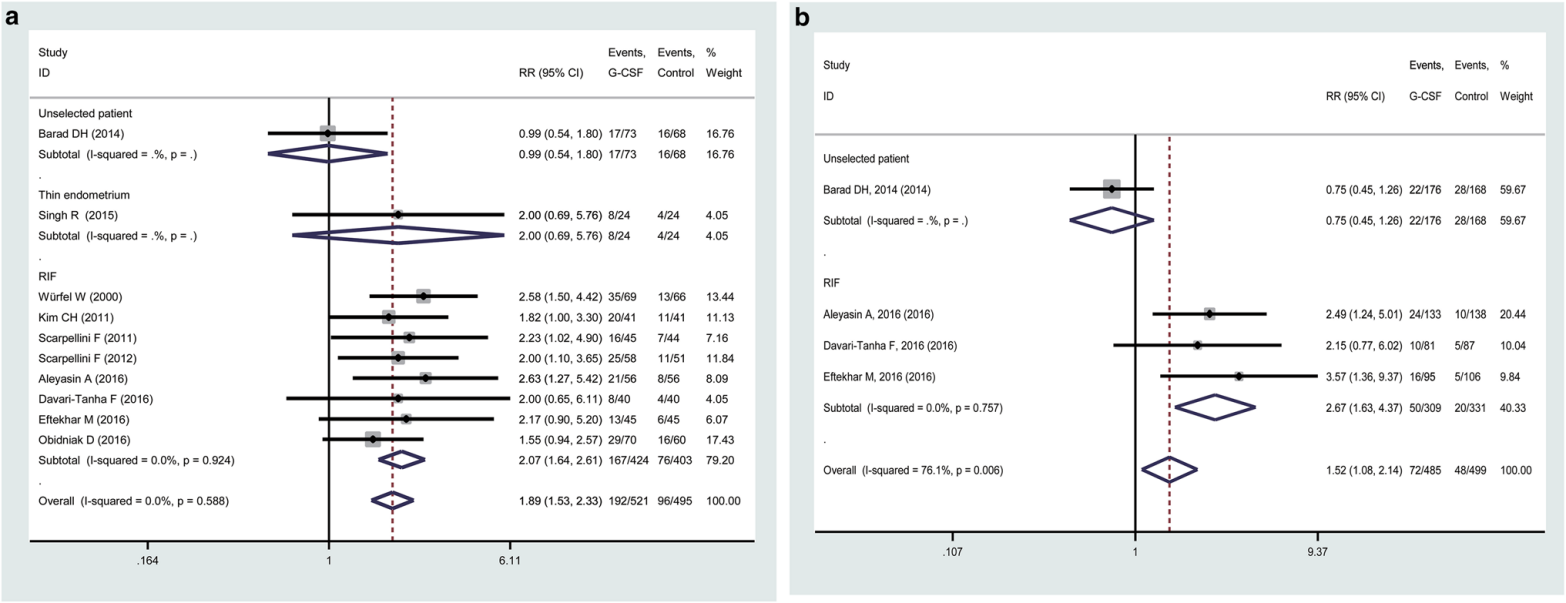
Forest plot comparing the effect of G-CSF on CPR (**a**) and IR (**b**) in infertile women undergoing IVF/ICSI for different indications of administration. Horizontal lines indicate 95% CIs; boxes show the study-specific weight; diamond represents combined effect size; dashed line indicates the overall estimate

To data, bulks of studies have explored the benefit of G-CSF therapy for cases with RIF or unresponsive thin endometrium, or unselected patients. However, few conclusive answers can be drawn from these reports, partly due to the modest sample size, heterogeneity of administration indications or included subjects, study design, or ethnicity. Therefore, a meta-analysis is expected to provide us with more reliable and comprehensive results (Fig. 4).

Previous pooled analysis have suggested that G-CSF administration may do some good for clinical outcomes after ART treatment, however, it is still unclear which specific conditions of infertility or through which administration route does the G-CSF treatment play an beneficial role. Zhao et al. first reported the meta-analysis on this topic [32], and found an improvement in PR after G-CSF administration for infertile cases with RIF and thin endometrium, while none change in IR. Additionally, it seems that only via subcutaneous route can G-CSF administration play its beneficial role, which is contrast to the conclusion by Xie et al. [33] that only through local perfusion can G-CSF treatment improve the clinical outcome (including endometrial thickness, clinical pregnancy rate, and embryo implantation rate) after ART treatment. Another inconsistence existed in the effect of increasing endometrial thickness, while Xie et al. [33] found a statistical significance, Li et al. [34] failed to get this findings, despite that both analyses show an obvious improvement in the clinical outcome.

After quality evaluation of previous meta-analysis, it can be found that studies included for pooled analysis involved in a broad range of designs, of which the observational is the majority. Case control, cohort study, or case analysis may incur relatively greater selective bias, report bias, or confounding as compared to that of RCT. This might impair the robustness of these pooled analyses, and result in the indeterminacy in conclusion as mentioned above. Well-designed RCTs have a stronger power to control considerable biases above, and may supply a relative robust outcome. Unfortunately, the disputes still remained among available data from single RCT.

Our analysis took on a total of 10 RCTs between 2000 and 2016, involving 1016 IVF-ET cycles (521 distributed to G-CSF group, and 495 to the control), with the average ages between 31 and 39 years. From this analysis, we can conclude that G-CSF administration is able to significantly improve the CPR in total population, but it unexpectedly does not do any good for embryo implanting, the same result to the study of Zhao et al. [32]. The paradox between the roles on CPR and IR can be explained by the limited number of included studies reporting IR data (*n* = 4). Moreover, a significant between-study heterogeneity existed in IR analysis, and was difficult to be traced due to too few studies included (*n* < 5). Therefore, we must be still cautious to deal with the conclusion regarding the role on IR. To the best of our knowledge, this is the first meta-analysis pooling the data from RCTs which investigate whether G-CSF does any good for IVF patients.

We also conducted a subgroup analysis by drug route and indication of G-CSF treatment, two important factors that should be considered emphatically. The pooled analysis sub-grouped by drug route came to a conclusion that both systematic administration and local perfusion of G-CSF be beneficial for ART treatment, which is partly inconsistent with Zhao et al.’s findings [32] that uterine infusion be not an efficient route of G-CSF administration. However, another meta-analysis by Xie et al. [33] support our viewpoint that intrauterine administration can improve the clinical outcome after embryo transfer. Through more detailed comparison, it can be believed that the conclusion of our and Xie’s analysis may be more reliable due to more studies included and more strict inclusion criterion on study design or patient type.

Among all RCTs included in our analysis, most were aimed at the cases with RIF (*n* = 8) and suggested a substantial efficiency of G-CSF treatment after pooled analysis, while only one at thin endometrium or unselected patients in each, and neither find the beneficial effect of GSF treatment. As to the cases with thin endometrium, two previous pooled analyses have indicated that these patients may benefit from G-CSF administration, however, almost all data were derived from observational studies, and the evidence not robust enough [33, 34]. Therefore, more RCTs are still needed to clear the therapeutic effect of G-CSF on thin endometrium cases.

To data, though various therapeutic propositions for G-CSF have already been reported, the specific molecular pathways of its endometrial and embryonic action have not yet been clear. It is generally accepted that establishment and maintenance of an intrauterine immune tolerance is an integral part of maternal–fetal interface, which is requisite for successful embryo implantation [35]. The mechanism underling this immunotolerance involved a T cell helper 2 (Th-2) dominant state and Treg cell proliferation [35–37]. G-CSF has been proven as a novel mediator of T cell tolerance to target at Th-2 and Treg cell [38] and play a critical role in regulation of the intrauterine immunotolerance [12, 39]. Despite limited evidence, regulating embryo development and endometrial vascular remodeling may be another two physiological roles of G-CSF as suggested by an in vitro blastocyst formation and endometrial ex vivo model test, respectively [13, 14]. Nevertheless, all above proof is just weak and preliminary, and most solitary, and increasing fundamental knowledge is expected to support the clinical applications of G-CSF in reproductive medicine.

Totally, this study is the first meta-analysis based on RCTs dealing with the role of G-CSF administration on clinical outcomes after embryo transfer. And we think the results are reliable as showed by the sensitivity and influence analysis. In conclusion, both systematic administration and local perfusion of G-CSF play a beneficial role in ART treatment, especially for the cases with RIF, but its role on the thin endometrium remains blur because insufficient data on these cases can be retrieved. Additionally, a little data about the rate of live birth can be extracted from included studies, which may impair the convincingness of this analysis.

## Electronic supplementary material

Below is the link to the electronic supplementary material.

Fig S1 Egger’s publication bias plots for comparing the effect of G-CSF on CPR (a) and IR (b) in infertile women undergoing IVF/ICSI (JPEG 2995 kb)

Fig S2 Influence analysis of individual study on the pooled estimate for CPR (a) and IR (b). Open circle indicates the pooled OR, given named study is omitted. Horizontal lines represent the 95% CIs (JPEG 3727 kb)
