# Supplementary figures and images for: Therapeutic role of granulocyte colony-stimulating factor (G-CSF) for infertile women under in vitro fertilization and embryo transfer (IVF-ET) treatment: a meta-analysis
Source: Arch Gynecol Obstet. 2018 Sep 15;298(5):861–71. doi: 10.1007/s00404-018-4892-4 (PMC6182707; doi:10.1007/s00404-018-4892-4)

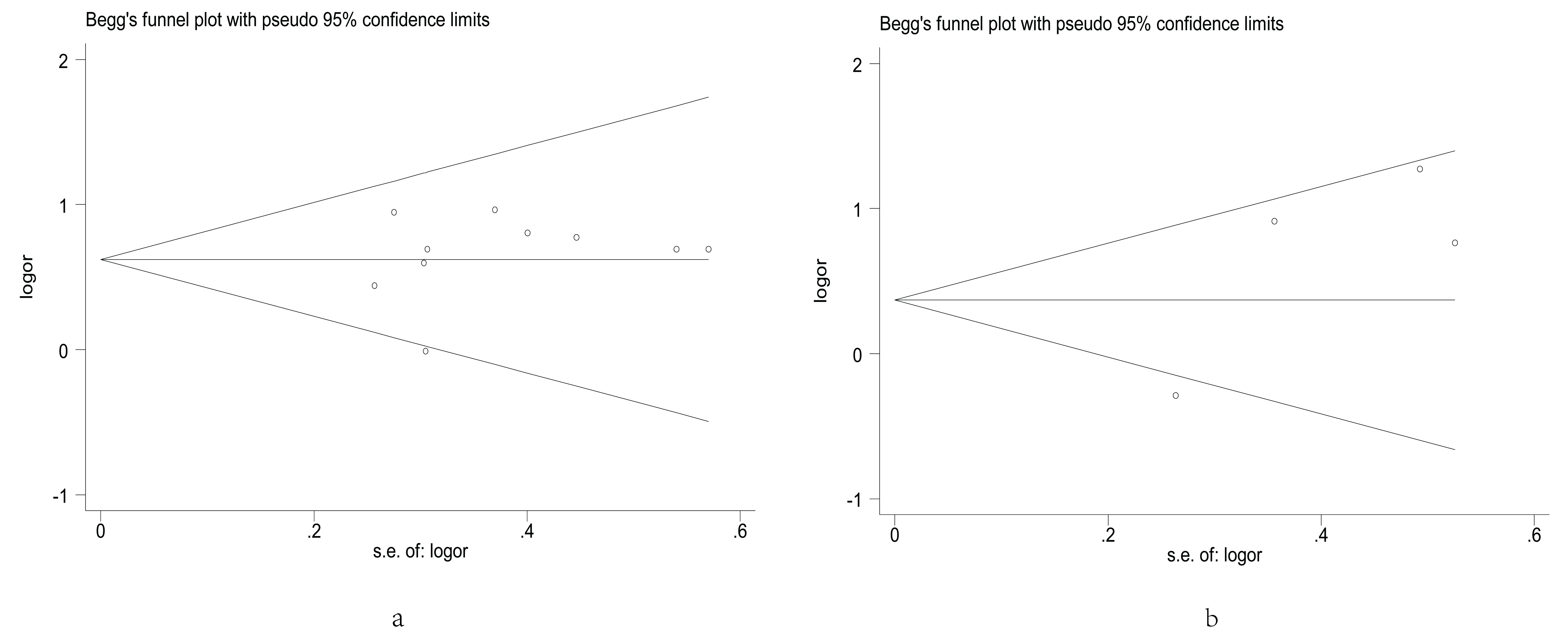

Supplement: Supplementary file 1 — Fig S1 Egger’s publication bias plots for comparing the effect of G-CSF on CPR (a) and IR (b) in infertile women undergoing IVF/ICSI (JPEG 2995 kb) [file 404_2018_4892_MOESM1_ESM.jpg]

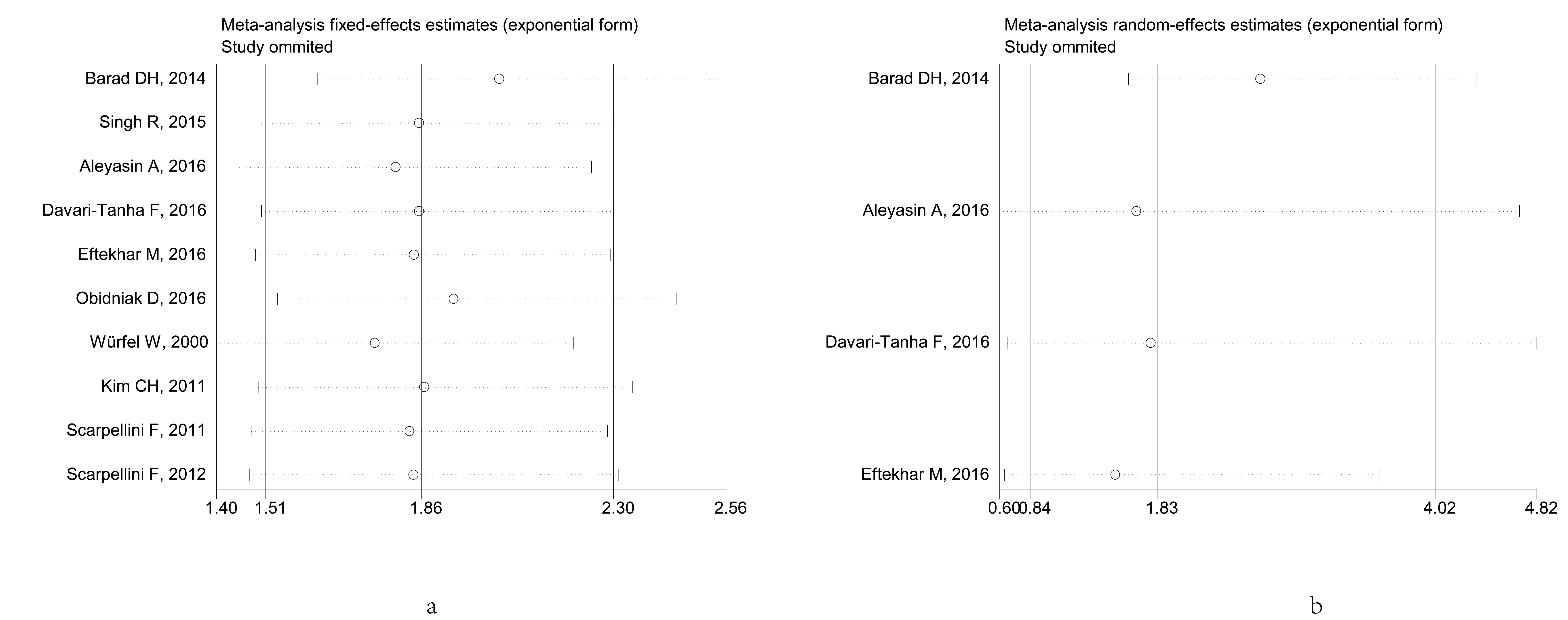

Supplement: Supplementary file 2 — Fig S2 Influence analysis of individual study on the pooled estimate for CPR (a) and IR (b). Open circle indicates the pooled OR, given named study is omitted. Horizontal lines represent the 95% CIs (JPEG 3727 kb) [file 404_2018_4892_MOESM2_ESM.jpg]
